# Changes in benzoxazinoid contents and the expression of the associated genes in rye (*Secale cereale* L.) due to brown rust and the inoculation procedure

**DOI:** 10.1371/journal.pone.0233807

**Published:** 2020-05-29

**Authors:** Magdalena Święcicka, Marta Dmochowska-Boguta, Wacław Orczyk, Agnieszka Grądzielewska, Anna Stochmal, Mariusz Kowalczyk, Leszek Bolibok, Monika Rakoczy-Trojanowska

**Affiliations:** 1 Department of Plant Genetics, Breeding and Biotechnology, Institute of Biology, Warsaw University of Life Sciences (SGGW), Warsaw, Poland; 2 Department of Genetic Engineering, Plant Breeding and Acclimatization Institute–National Research Institute, Radzików, Błonie, Poland; 3 Department of Horticultural Plant Genetics and Breeding, Institute of Plant Genetics, Breeding and Biotechnology, University of Life Sciences in Lublin, Lublin, Poland; 4 Department of Biochemistry and Crop Quality, Institute of Soil Science and Plant Cultivation—State Research Institute, Puławy, Poland; 5 Department of Forest Silviculture, Institute of Forest Sciences, Warsaw University of Life Sciences (SGGW), Warsaw, Poland; National Institute of Technology Rourkela, INDIA

## Abstract

Benzoxazinoids (BXs) are secondary metabolites with diverse functions, but are primarily involved in protecting plants, mainly from the family Poaceae, against insects and fungal pathogens. Rye is a cereal crop that is highly resistant to biotic stresses. However, its susceptibility to brown rust caused by *Puccinia recondita* f. sp. *secalis* (*Prs*) is still a major problem affecting its commercial production. Additionally, the genetic and metabolic factors related to this disease remain poorly characterized. In this study, we investigated whether and to what extent the brown rust infection and the inoculation procedure affect the contents of specific BXs (HBOA, GDIBOA, DIBOA, GDIMBOA, DIMBOA, and MBOA) and the expression of genes related to BX (*ScBx1–5*, *ScIgl*, and *Scglu*). We revealed that treatments with water and a urediniospore suspension usually downregulate gene expression levels. Moreover, HBOA and DIBOA contents decreased, whereas the contents of the remaining metabolites increased. Specifically, the MBOA content increased more after the mock treatment than after the *Prs* treatment, whereas the increase in GDIBOA and GDIMBOA levels was usually due to the *Prs* infection, especially at two of the most critical time-points, 17 and 24 h post-treatment. Therefore, GDIBOA and GDIMBOA are glucosides that are important components of rye defence responses to brown rust. Furthermore, along with MBOA, they protect rye against the stress associated with the inoculation procedure used in this study.

## Introduction

Benzoxazinoids (BXs) are secondary metabolites synthesized mainly by species belonging to the family Poaceae, including rye (*Secale cereale* L.). Several studies have confirmed that BX biosynthesis comprises several steps [1–6]. First, indole-3-glycerol phosphate is converted to indole, which is then transformed to indolin-1-one. Next, three monooxidations result in the synthesis of 2,4-dihydroxy-1,4-benzoxazin-3-one (DIBOA). Other reactions include the glycosylation of DIBOA to produce 2-O-β-glucoside (GDIBOA) and O-methylation to generate 2,4,7-trihydroxy-1,4-benzoxazin-3-one glucoside (GTRIBOA) as well as the glycosylation of 2,4-dihydroxy-7-methoxy-1,4-benzoxazin-3-one (DIMBOA) to produce 2,4-dihydroxy-7-methoxy-1,4-benzoxazin-3-one glucoside (GDIMBOA) and O-methylation to generate 4,7-dimethoxy-2-{[3,4,5-trihydroxy-6-(hydroxymethyl)oxan-2-yl]oxy}-3,4-dihydro-2H-1,4-benzoxazin-3-one glucoside (GHDMBOA). Hydroxylations convert GDIBOA and GDIMBOA to DIBOA and DIMBOA, respectively.

Several genes controlling BX biosynthesis have been isolated and sequenced. The species with the most characterized *Bx* genes is maize [2, 4–6]. In rye, nine genes, *ScBx1–ScBx7*, *ScGT*, and *Scglu* reportedly control reactions corresponding to most of the BX biosynthesis reactions determined in maize [7–12]. Recently, Rakoczy-Trojanowska *et al*. [13] and Wlazło *et al*. [14] proved that another gene, *ScIgl*, has the same function as *ScBx1* in late developmental stages.

Benzoxazinoids are primarily important components of plant defence strategies against biotic and abiotic stresses. They may also help regulate flowering time, auxin metabolism, iron uptake, and aluminium tolerance [15] as well as control root–microbe interactions via a global regulatory function related to root secondary metabolism [16]. Although the role of BXs in defence against insects has been broadly studied and well documented, their association with disease resistance is not obvious [1–3]. Additionally, the correlation between BX content and composition and disease resistance may depend on the infection site and pathogen characteristics. For example, in the case of northern corn leaf blight caused by the hemi-biotrophic fungal pathogen *Helmisthosporium turcicum*, the BX concentration of the midstalk leaves is positively correlated with resistance and negatively–with symptom development [17]. Similarly, in wheat seedlings, reactions to stem rust and head blight are negatively correlated with BX contents [18]. In contrast, maize responses to anthracnose caused by *Colletotrichum graminicola* are not correlated with the BX concentration [19]. As in the case of the passive defence mechanism, the induced BX-based defence is also ambiguous and determined by specific factors (e.g., plant–pathogen interactions and pathogen virulence). Ahmad *et al*. [20] reported that an infection by *Exserohilum turcicum* stimulates the accumulation of apoplastic BX during the early infection stages. Moreover, Song *et al*. [21] proved that the inoculation of maize with the arbuscular mycorrhizal fungus *Glomus mosseae*, which minimizes the symptoms of sheath blight triggered by the necrotrophic fungus *Rhizoctonia solani*, significantly increases the DIMBOA content in the roots, whereas an inoculation with *R*. *solani* alone does not have a similar effect. Furthermore, an infection by the necrotroph *Septoria tritici* results in the hydrolysis of DIMBOA glucoside, whereas an infection by *Drechslera teres*, which is a necrotroph incompatible with wheat, only slightly decreases the DIMBOA glucoside concentration, and an infection by the obligate parasite *Puccinia recondita* does not alter the DIMBOA glucoside concentration [22]. Yang *et al*. [23] reported that the resistance to northern corn leaf blight is associated with a decrease in the abundance of BX secondary metabolites.

Brown rust (BR) of rye, which is caused by the obligate biotrophic basidiomycete *P*. *recondita* f. sp. *secalis* (*Prs*) (Roberge ex Desmaz), is one of the most important diseases of rye in Central and Eastern Europe [24, 25]. Yield losses due to BR can be up to 40% under natural conditions [26], but can be as high as 80% following an early infection [27]. Several rye *R* genes (i.e., *Pr* genes) associated with defence responses to *Prs* have been described, including *Pr1–5*, *Pr-d–f*, *Pr-i–l*, *Pr-n*, and *Pr-p–t*, all of which are dominantly inherited [28].

There is no available information regarding whether BXs influence BR resistance. Moreover, how a BR infection affects BX synthesis, including the expression of the associated genes, remains relatively unclear. Nevertheless, Rakoczy-Trojanowska *et al*. [29] suggested a possible relationship between BX and BR. Specifically, they determined that a single nucleotide polymorphism (ScBx4_1583) in *ScBx4* (encoding a cytochrome P450 monooxygenase) is stably associated with BR resistance.

The objective of this study was to determine whether a *Prs* infection and the subsequent disease development as well as the inoculation procedure itself influence the expression levels and profiles of the following seven genes: *ScBx1–ScBx5*, *ScIgl*, and *Scglu*, of which, the first six control the production of six BXs [2-hydroxy-4H-1,4-benzoxazin-3-one (HBOA), GDIBOA, DIBOA, GDIMBOA, DIMBOA, and 6-methoxybenzoxazolinone (MBOA)] and the last one mediates the hydroxylation of glucosides to aglucones. We were also interested in clarifying if the BX content is associated with the plant–pathogen interaction at a given time-point.

## Results

### Analysis of pathogenesis in a preliminary experiment

**Fig 1:**
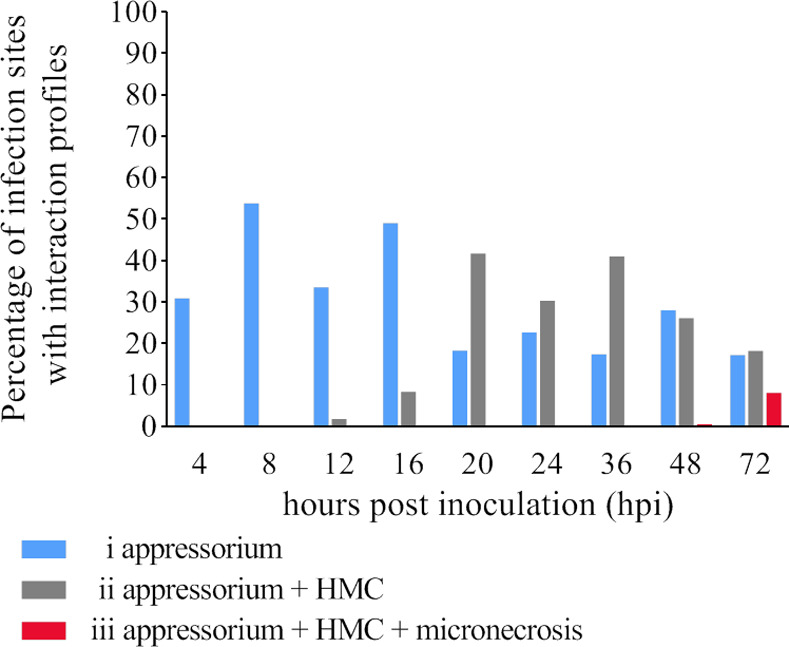
Plant–pathogen profiles i, ii, and iii on susceptible rye cultivar Słowiańskie inoculated with single spore of *Puccinia recondita*. Leaf samples were collected 4, 8, 12, 16, 20, 24, 36, 48 and 72 hours post inoculation (hpi) and calcofluor white stained.

**Fig 2:**
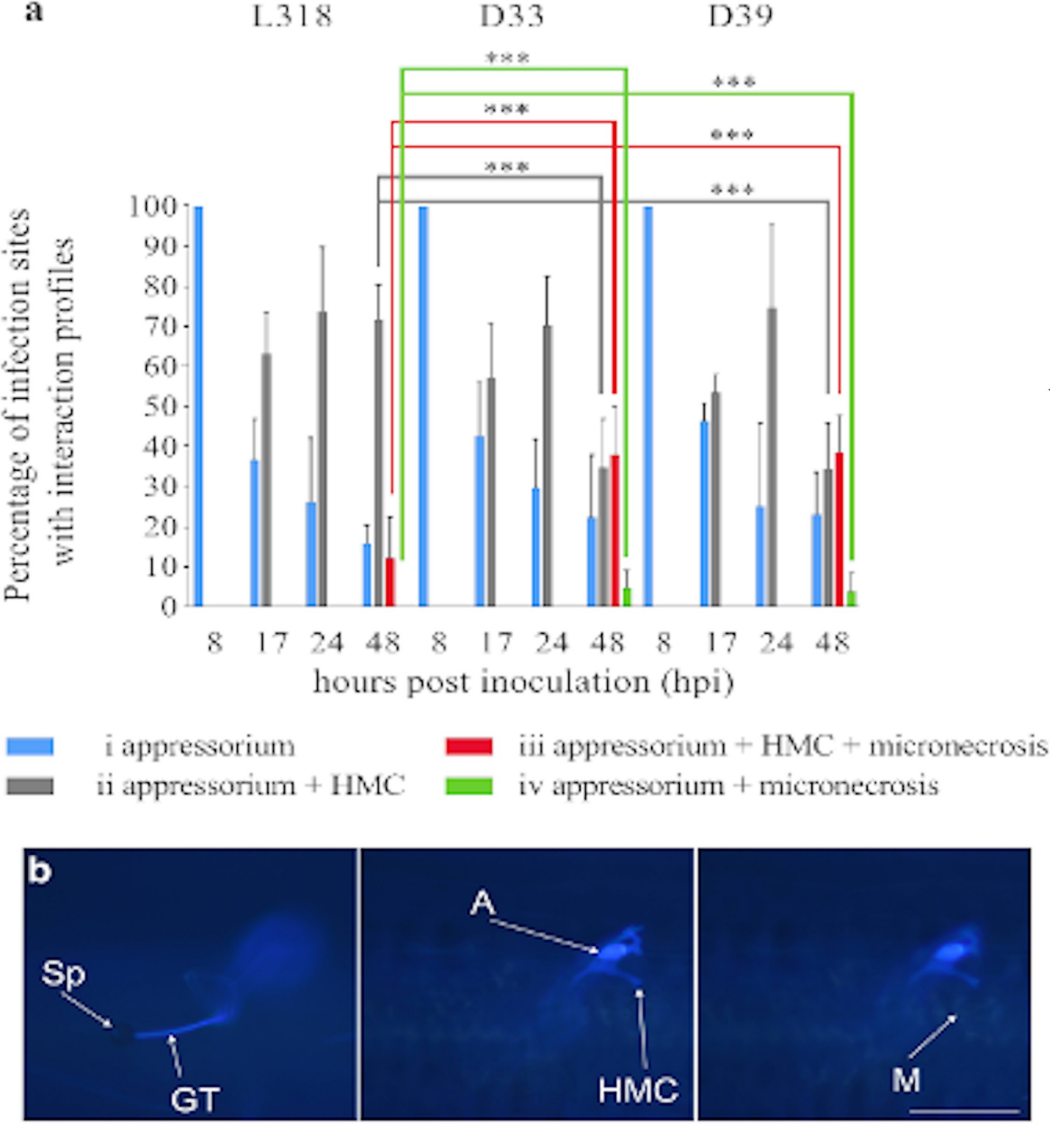
(A) Plant–pathogen interaction profiles for the seedlings of rye inbred lines: L318, D33, and D39. The data represent mean and standard derivation. *** p < 0.001 according to the ANOVA and LSD *post-hoc* tests. (B) Example of profile iii in line D33 at 48 hours post inoculation (hpi) as determined with a fluorescence microscope. Sp: spore; GT: germ tube; A: appressorium; HMC: haustorial mother cell; M: micronecrosis. Bar = 100 μm.

Four plant–pathogen interaction profiles were observed during the disease development following the inoculation of rye cv. Słowiańskie (Fig 1). Profile i (infection site with developed appressoria indicating an effective pathogen infection) was assigned to 30.9% of the infection sites on leaves collected at 4 h post-inoculation (hpi) and 53.8% of the infection sites at 8 hpi. Profile ii [developed haustoria mother cells (HMCs) indicating the advancement of infection stages] was detected in 1.8%, 8.4%, and 41.7% of the samples at 12, 16, and 20 hpi, respectively. Profile iii (micronecrosis symptoms due to the plant resistance response) (Fig 2B) was detected for 0.5% and 8.1% of the infection sites at 48 and 72 hpi, respectively. None of the infection sites were assigned as profile iv (micronecrosis symptoms without HMCs).

### Analysis of pathogenesis in the main experiment

All infection sites were designated as profile i at 8 hpi (Fig 2A). Profiles i and ii were detected at 17 and 24 hpi. Despite the very similar responses in the detached leaf test, at the seedling stage, the lines differed from each other in terms of plant–pathogen interaction profiles. Specifically, the percentage of infection sites designated as profile i for L318, D33, and D39 was 36.8% (standard deviation, sd = 10.2), 42.70% (sd = 13.3), and 46.4% (sd = 4.5), respectively, at 17 hpi and 26.2% (sd = 16.1), 29.7% (sd = 12.2), and 25.2% (sd = 20.7), respectively, at 24 hpi. The percentage of infection sites scored as profile ii for L318, D33, and D39 was 63.2% (sd = 10.2), 57.3% (sd = 13.3), and 53.6% (sd = 4.5), respectively, at 17 hpi and 73.8% (sd = 16.1), 70.3% (sd = 12.2), and 74.8% (sd = 20.7), respectively, at 24 hpi. All four profiles were observed at 48 hpi. At this time-point, similar profile i rates were observed for all lines (16%, sd = 4.4; 22.4%, sd = 15.5 and 23.1%, sd = 10.6 for L318, D33, and D39, respectively). Additionally, the profile ii rates for lines D33 and D39 (34.8%, sd = 12.2 and 34.4%, sd = 11.6, respectively) were approximately half of that of line L318 (71.9%, sd = 8.5). The profile iii rate was similarly high in lines D33 (38.1%) and D39 (38.6%), but was much lower in line L318 (12.2%, sd = 10.2). Profile iv was observed for only D33 and D39 (4.8%, sd = 4.2 and 3.8%, sd = 4.6, respectively).

**Fig 3:**
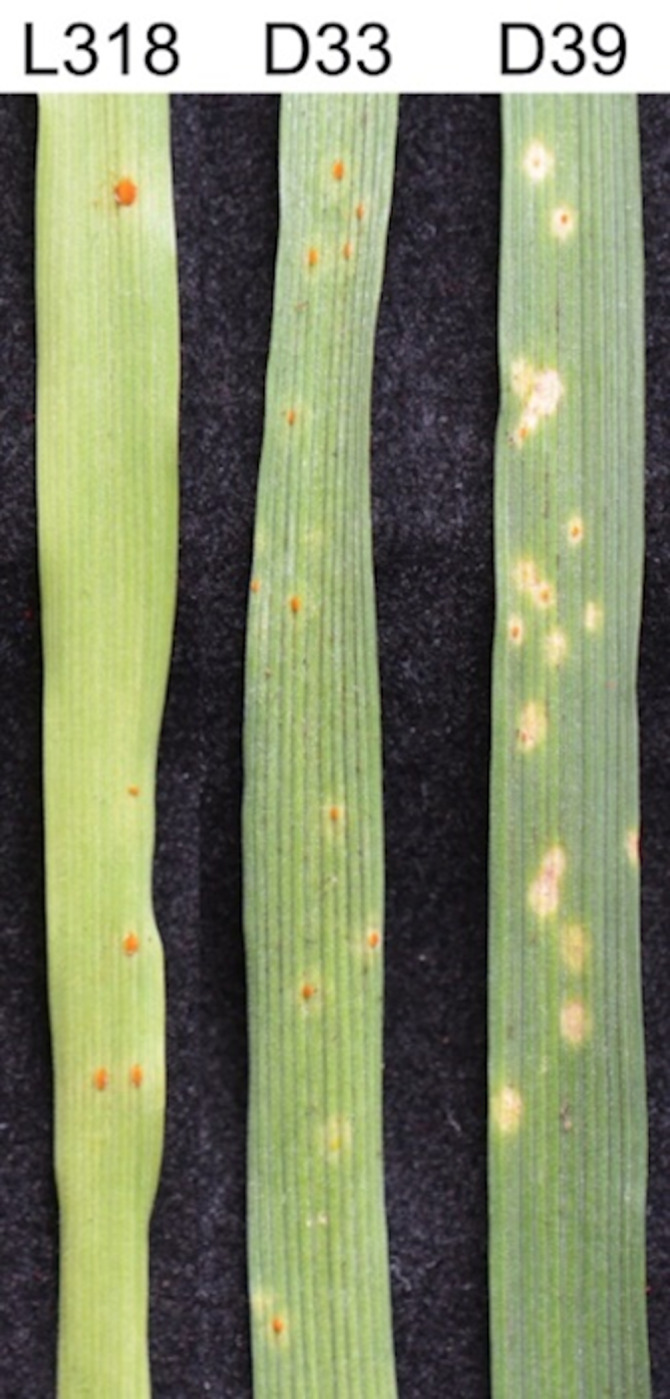
Macroscopic disease symptoms on seedlings of three rye ILs: L318, D33, and D39 inoculated with *Puccinia recondita* (*Prs*) (isolate No. 1.1.6) urediniospores at 15 dpi.

The infection type of inoculated lines L318, D33, and D39 was scored as 3 (medium-sized uredinia with chlorosis), 2 (medium-sized uredinia with necrosis), and 1 (small uredinia with necrosis), respectively. Accordingly, of the analysed lines, L318 was the most susceptible to the *Prs* infection (Fig 3) which is consistent with the results obtained previously for plants grown in the field (S9 Table).

### Dissecting the effect of the treatment procedure

#### Influence of the treatment with water and an aqueous suspension of *Prs* urediniospores on gene expression levels

**Fig 4:**
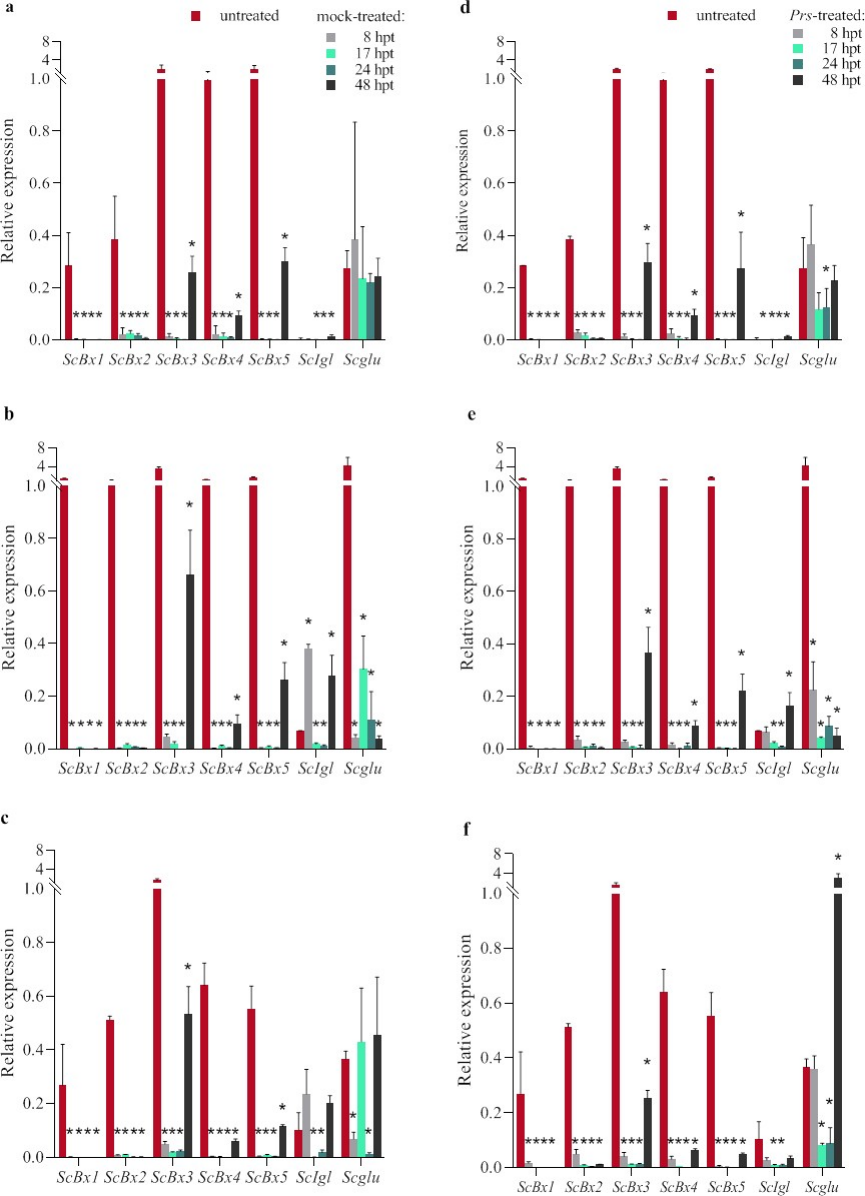
Expression patterns of *ScBx1–5*, *ScIgl*, and *Scglu* genes in untreated, mock-treated (abc), and *Prs*-treated (def) plants of rye ILs L318 (ad), D33 (be), D39 (cf) at four examined time-points, 8, 17, 24, and 48 hpt. The data represent mean value with standard derivation, * indicates statistically significant at p < 0.05 (based on the Mann-Whitney U test).

In most cases, the early [8 h post-treatment (hpt)] response of *ScBx1–5* to the mock and *Prs* treatments was a decrease in expression levels (relative to the corresponding expression levels of the untreated plants) (Fig 4 and S1–S3 Tables). Regarding *ScIgl* and *Scglu*, the water and *Prs* urediniospore treatments either decreased or increased their expression levels at 8 hpt, but a significant increase with the untreated control level was detected for only the mock-treated D33 seedlings. Usually, the decrease in gene expression after the first 8 hpt was greater for the seedlings infected with *Prs* than for the mock-treated seedlings.

At subsequent time-points, the gene expression levels remained generally unchanged (especially *ScBx1* and *ScBx2*) (Fig 4). However, the following three patterns of temporal gene expression changes were observed (with a total frequency of 45.2%): (1) similar level at two subsequent time-points and an increase at 48 hpt (the most frequent pattern); (2) further decrease up to 17 hpt and/or 24 hpt, followed by an increase at the last time-point; and (3) alternating increase and decrease. The greatest increase in expression level (more than 6-fold compared with the initial level) was detected for *Scglu* in the *Prs*-treated seedlings of the most resistant line, D39, at 48 hpt (Fig 4F). The greatest decrease in expression level was observed for *Scglu* in line D33 after the mock and *Prs* treatments at all time-points (Fig 4B and 4E). With a few exceptions, the greatest increase in gene expression was observed at the last time-point.

#### Influence of the treatment with water and an aqueous suspension of *Prs* urediniospores on the BX content

**Fig 5:**
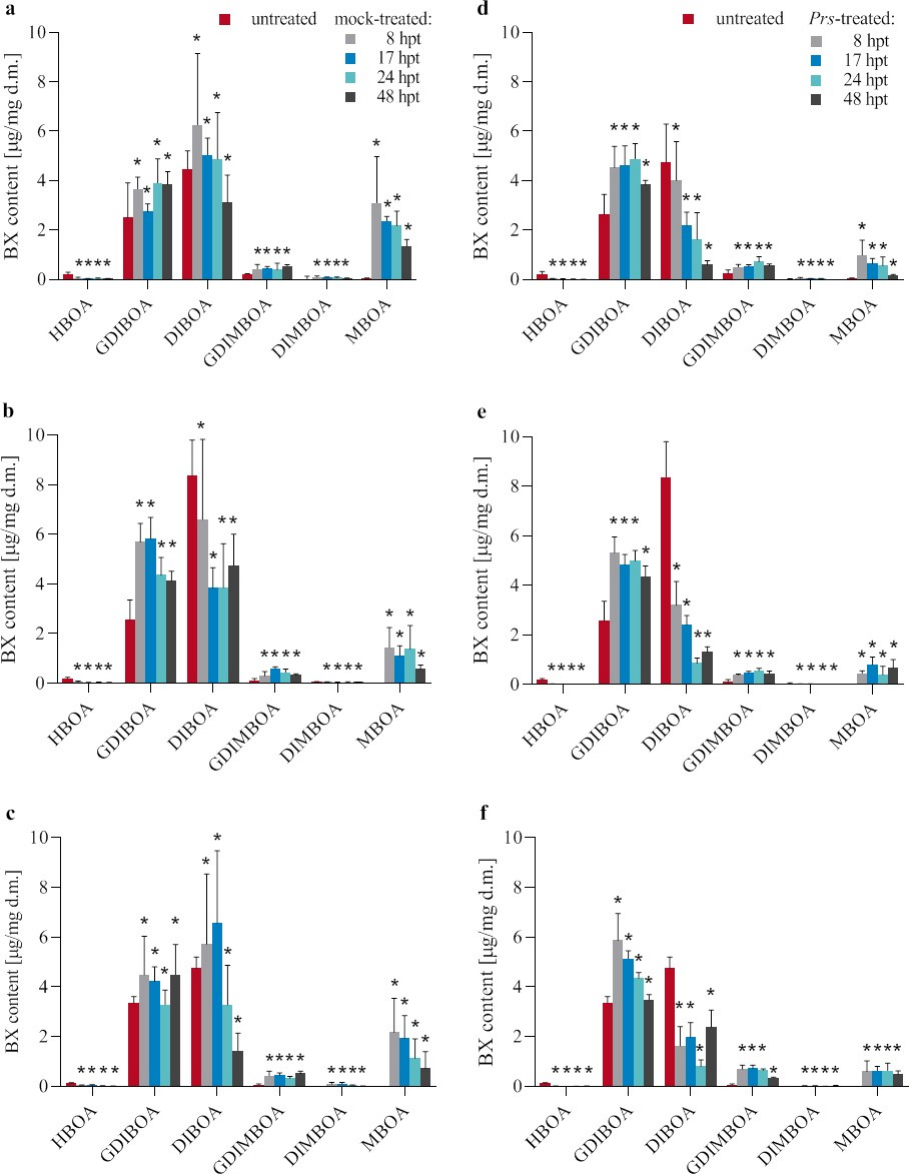
Synthesis patterns of BXs in untreated, mock-treated (abc) and *Prs*-treated (def) plants of rye ILs L318 (ad), D33 (be), D39 (cf) at four examined time-points, 8, 17, 24, and 48 hpt. The data represent mean value with standard derivation, * indicates statistically significant at p < 0.05 (based on the Mann-Whitney U test).

In contrast to the gene expression changes, the BX contents usually increased at 8 hpt for the mock- and *Prs*-treated seedlings (relative to the BX contents of the untreated plants) (Fig 5 and S4–S6 Tables). A clear decrease in content was observed for HBOA in all three lines, for DIBOA and DIMBOA in line D33 subjected to mock and *Prs* treatments, and for DIBOA in lines L318 and D39 treated with *Prs*. At the first time-point, the greatest increase in BX contents was observed for MBOA in mock-treated L318 seedlings (Fig 5A). The largest decrease in the DIBOA content was detected in the *Prs*-treated D33 seedlings (Fig 5E).

During the next 40 h, the BX level remained relatively unchanged in a few cases. However, the following five BX content profiles were more frequently detected (61.1%): (1) continued decrease; (2) an increase at 17 hpt and/or 24 hpt, followed by a decrease at the last time-point; (3) stable level up to 24 hpt, followed by a decrease at 48 hpt; (4) stable level up to 24 hpt, followed by an increase at 48 hpt; and (5) alternating increase and decrease at subsequent time-points. With one exception each, the following five BXs were more affected by *Prs* than the mock treatment: GDIBOA, DIBOA, GDIMBOA, DIMBOA, and especially HBOA. The greatest increase in the BX content (relative to the initial level) was detected for GDIBOA in the mock-treated D33 seedlings at the second time-point (Fig 5B), whereas the greatest decrease in the BX content was observed for DIBOA in the *Prs-*treated D33 seedlings at the third time-point (Fig 5E). In contrast, the MBOA content was higher in the seedlings treated with water than in the *Prs-*treated seedlings.

### Dissecting the effect of brown rust

#### Influence of the *Prs* infection on gene expression

**Fig 6:**
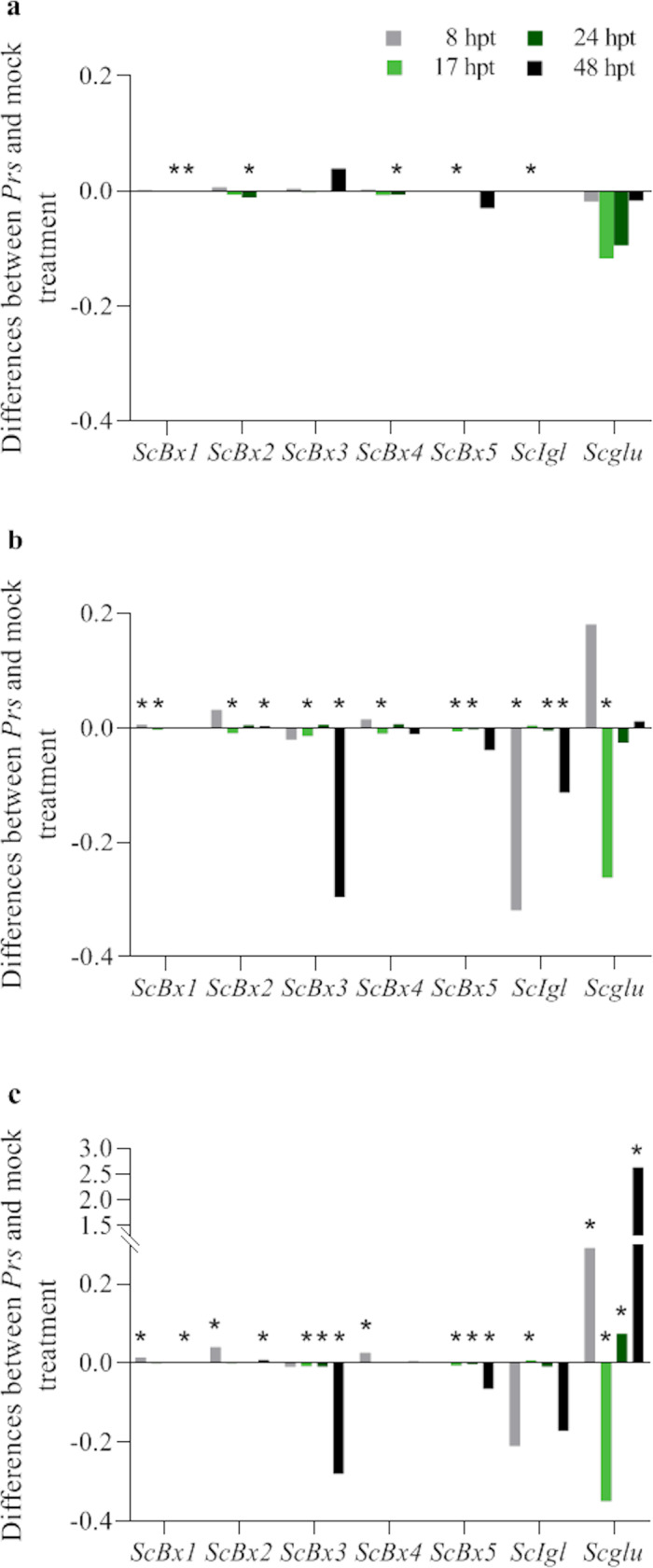
Differences in the *ScBx1*–*5*, *ScIgl*, and *Scglu* expression levels between *Prs*- and mock-treated rye IL L318 (a), D33 (b), and D39 (c) plants. The data represent mean value with standard derivation, * indicates statistically significant at p < 0.05 (based on the Mann-Whitney U test).

A comparison of the gene expression in mock- and *Prs*-treated plants at 8, 17, 24, and 48 hpt revealed that in most cases (58.3%), the infection decreased expression levels (Fig 6 and S7 Table). On average, 41.6% of the differences were significant. Specifically, 21.4%, 46.4%, and 57.1% of the differences were significant for lines L318, D33, and D39, respectively. Among the positive differences, 31.4% were significant, with 0.0%, 18.2%, and up to 60% of the positive differences confirmed as significant for lines L318, D33, and D39, respectively. The line with the most genes exhibiting upregulated expression was D39, especially at the first time-point (Fig 6C). In line D33, upregulated expression was observed only for *ScBx1* at 8 hpt and *ScBx2* at 48 hpt (Fig 6B). None of the analysed genes had significantly upregulated expression levels in line L318 following the *Prs* infection (Fig 6A). Of the three investigated lines, D39 was the most responsive, with *Prs* upregulating and downregulating expression levels (depending on the gene and time-point). Line D33 exhibited a moderate response level to *Prs*, whereas L318 exhibited the weakest response. The intensity of the reactions corresponded with the infection types. The expression levels of *ScBx3* in D39, *ScBx5* in D33, and *Scglu* in L318 only decreased, but the upregulated or downregulated expression of the remaining genes in response to *Prs* depended on the rye genotype and the time after the infection. The greatest increases and decreases in expression levels were determined for *Scglu* in line D39 at 17 and 48 hpt, respectively (Fig 6C).

#### Influence of the *Prs* infection on the BX contents

**Fig 7:**
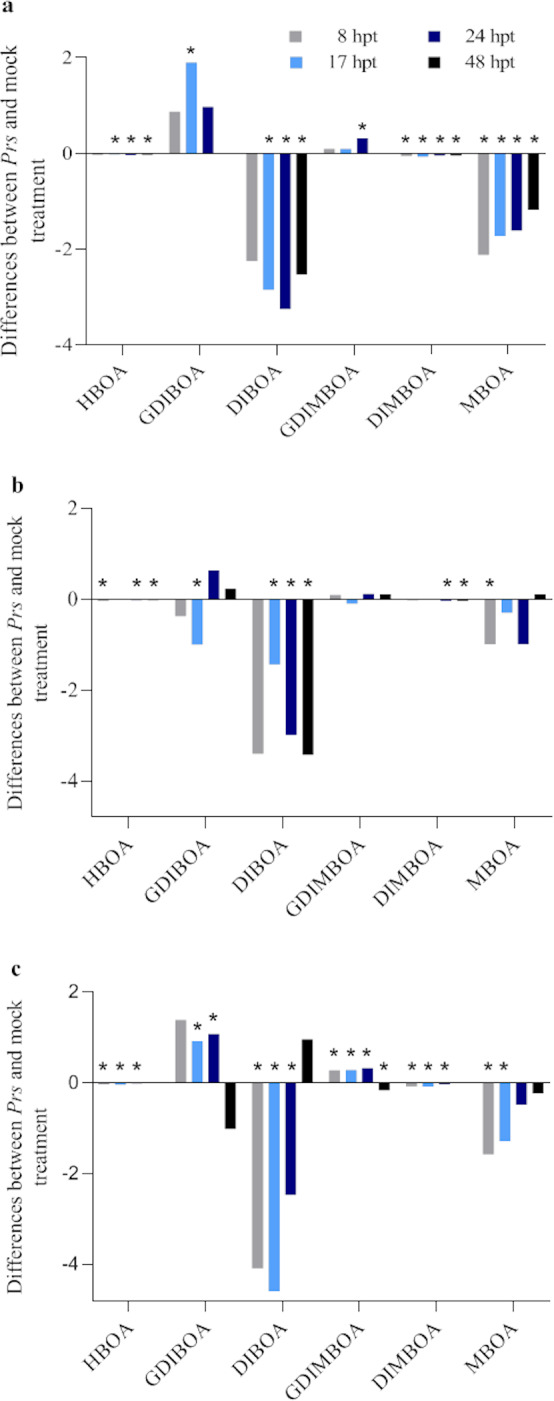
Differences in the BX synthesis levels between *Prs*- and mock-treated rye IL L318 (a), D33 (b), and D39 (c) plants. The data represent mean value with standard derivation, * indicates statistically significant at p < 0.05 (based on the Mann-Whitney U test).

The infection of rye plants with *Prs* affected the contents of most BXs, with decreases rather than increases more commonly observed (70.8%) (Fig 7 and S8 Table). Among the negative differences, 70.6% were significant, with 82.4%, 55.6%, and 75.0% of these differences revealed as significant in lines L318, D33, and D39, respectively. One-third of the positive differences were significant, with 28.6%, 0.0%, and 62.5% of these differences confirmed as significant in L318, D33, and D39, respectively.

The most and least responsive lines were D39 and D33, respectively. The time-points with the most changes in BX contents and the most cases of increased contents were 17 and 24 hpt. The GDIBOA and GDIMBOA contents usually increased in response to *Prs*, whereas the opposite pattern was observed for the other examined BXs. Generally, similar reactions to *Prs* were observed in all lines. Specifically, at a given time-point, the contents of a BX either increased or decreased in all lines. An exception was GDIBOA in D33. Relative to the corresponding level in the mock-treated control, the GDIBOA content in D33 decreased at the first and second time-points, but increased at 24 and 48 hpt (Fig 7B). However, in lines L318 and D39, the GDIBOA contents increased at the first three time-points and then decreased (Fig 7A and 7C). Overall, the greatest increase in BX content was detected for GDIBOA in line L318 at 17 hpt (Fig 7A), whereas the largest decrease in BX content was recorded for DIBOA in line D39 at 17 hpt (Fig 7C).

## Discussion

Plant secondary metabolites are associated with diverse processes, including resistance to pathogens. In some cereal species, mainly rye, wheat, and maize, BXs are the main class of secondary metabolites involved in responses to fungal pathogens [3,15]. Nevertheless, their role in protecting plants against diseases remains relatively uncharacterized [23]. Even less is known about the induced defence, especially regarding gene expression levels. Moreover, there are only a few reports describing the relationship between infections by biotrophic fungi and changes in BX biosynthesis and its regulation [e.g., 20, 23]. Accordingly, we decided to clarify if and how the synthesis of six BXs (HBOA, GDIBOA, DIBOA, GDIMBOA, DIMBOA, and MBOA) and the expression of seven genes (*ScBx1–5*, *ScIgl*, and *Scglu*) are affected by a BR infection, which is one of the most important rye diseases. Additionally, we were also interested in how the infection procedure *per se* influences both gene expression and metabolite synthesis.

The investigated genes included *ScBx1–5* as well as *ScIgl* and *Scglu*. The *ScIgl* gene has the same function as *ScBx1* in the late developmental stages [13,14]. The *Scglu* gene encodes a β-glucosidase that hydrolyses stable glucosides to reactive aglucones following the plant tissue destruction due to herbivores or pathogens [6–8, 30]. We assumed that the structural disorganization of plant cells caused by *Prs* during the formation of haustoria [31–33] might modify *Scglu* expression. This is consistent with the results published by Gomez-Anduro *et al*. [34], who proved that in maize, *Zmbglu2* expression is negatively influenced by mechanical damage. Additionally, changes to intracellular tensions caused by the formation of haustoria should lead to the release of glycosides from vacuoles and their interaction with β-glucosidases.

To determine the effects of treatments with water and an aqueous suspension of fungal spores on gene expression and BX contents, we compared the data obtained for mock- and *Prs*-treated plants with the corresponding data for the untreated plants. The effects of the mock and *Prs* treatments were compared at four time-points to clarify the influence of the pathogen infection on the gene expression and BX contents. To the best of our knowledge, such an approach that considers all elements of the infection procedure has not been applied in similar types of studies to date. Researchers have typically used two treatments (mock and pathogen treatments) [e.g., 35–37] or they have compared pathogen-infected plants with untreated plants [e.g., 38, 39]. Thus, they were unable to assess the impact of the treatment procedure, which is particularly important for analyses of secondary metabolism. Moreover, some of the conclusions of these earlier investigations may not be legitimate.

### Dissecting the effect of the treatment procedure

#### Influence of the treatment with water and an aqueous suspension of *Prs* urediniospores on gene expression

Considering the specificity of secondary metabolism, we assumed that the procedures for the mock treatment and infection with *Prs* include elements that most likely affect BX metabolism and the expression of the associated genes. The underlying mechanism may be associated with the micro-wounds caused by brushing, which may increase jasmonic acid (JA) levels and induce the BX defence pathway. The role of JA in BX biosynthesis has been well documented [40, 41].

The effects of the treatments in this study (regardless of whether the plants were sprayed with water or an aqueous suspension of urediniospores) were very noticeable. Regarding gene expression, the *Prs* infection and treatment with water most commonly resulted in downregulated expression levels, which were generally comparable for both stresses. With a few exceptions, the differences between the expression levels in the untreated and stressed plants at the first time-point (8 hpt) were slightly greater for the mock-treated plants, implying that in the early stage after the treatments, the changes in gene expression levels were primarily due to the treatment *per se* and not the pathogen. Our findings regarding *ScBx1* expression differ from those of a study by Ding *et al*. [36], in which the expression of maize *Bx1*, which is an orthologue of *ScBx1* [10], was usually upregulated in the shoots of plants treated with different elicitors from the pepper pathogen *Phytophthora capsici* and pepper root exudates to induce plant defence mechanisms at a similarly early time-point (12 hpt). During the next 40 h, the gene expression levels remained relatively stable. Only in slightly more than a quarter of the cases were other profiles observed. We conclude that the treatment procedures, regardless of whether the plants were mock- or *Prs*-treated, induce a strong and rapid reaction, but they do not cause further changes. Although we expected to observe additional changes as the BR infection progressed, they occurred only sporadically and were mainly associated with *ScIgl* expression at 48 hpt in lines L318 and D33. Similarly, Ahmad *et al*. [20] postulated that in maize, *Igl* expression is induced by stress. In the current study, downregulated *ScIgl* expression was most often observed. Additionally, in some cases (L318, D33, and D39 at 8 hpt), the mock treatment increased *ScIgl* expression, whereas the *Prs* infection had the opposite effect.

#### Influence of the treatment with water and an aqueous suspension of *Prs* urediniospores on BX contents

Only two BXs, HBOA and DIBOA (with few exceptions), underwent content changes that were similar to the gene expression changes. In contrast, completely different changes were observed for three other BXs, GDIBOA, GDIMBOA, and MBOA. Furthermore, the changes in the DIMBOA content depended on the genotype, with both stresses increasing the content in L318 and D39, but decreasing the content in D33. The *Prs* spore treatment usually resulted in a greater change to BX contents regardless of the rye genotype. The exception was the MBOA content, which increased considerably more in the mock-treated plants. On the basis of these observations, we hypothesize that three BXs, GDIBOA, GDIMBOA, and MBOA, are crucial components of rye defence reactions, with the first two (especially GDIMBOA) protecting plants against BR, and MBOA providing protection against the treatment procedure *per se*.

### Dissecting brown rust effects

#### Effect of the *Prs* infection on gene expression

To elucidate the effects of a fungal infection, the gene expression level differences between the mock- and *Prs-*treated plants were analysed at four time-points. The *Prs* infection mainly negatively influenced gene expression. However, the direction of the changes depended on the genes (i.e., *ScBx2* and *ScBx4* expression levels were more frequently upregulated, whereas *ScBx5* and *ScIgl* expression levels were downregulated), time-points (i.e., upregulated and downregulated expression levels were mostly detected at 8 and 17 hpt, respectively), and genotypes (i.e., most of the instances of upregulated expression occurred in D39). At the first time-point, the *ScBx1*, *ScBx2*, and *ScBx4* expression levels increased in all lines. Nevertheless Yang *et al*. [23] concluded there is a negative relationship between the expression levels of the maize *Bx1* and *Bx2* genes and resistance to northern corn leaf blight. This discrepancy may be explained by the fact the Yang *et al*. [23] study involved the fungus *E*. *turcicum*, which is characterized by a different etiology and is more invasive than *Prs*. Moreover, La Hovary [9] subjected rye seedlings to wounding and the reported *ScBx1* and *ScBx2* expression levels at 6 hpt are similar to our observed expression levels, but a JA treatment downregulated the expression of both genes (relative to the corresponding expression levels in the mock-treated plants). The frequency of upregulated expression levels corresponded with the defence reaction type, with the highest number of instances observed in the least susceptible line, D39. In contrast, the most susceptible line, L318, had no significantly increased expression levels. Additionally, D39 was the only line in which *ScBx1*, *ScBx2*, and *Scglu* expression levels were significantly upregulated in the infected plants in a coordinated manner at the first and last time-points. The *ScBx4* expression level increased in the infected D39 seedlings at all time-points. A published association analysis [29] revealed a significant role for *ScBx4* in the resistance of mature rye plants to BR under field conditions. In the current study, we confirmed the importance of this gene for the BR resistance in much younger rye plants, but only for the resistant genotype. Therefore, the previously identified polymorphism, ScBx4_1583, may be associated with non-race-specific adult plant and seedling stage resistance.

Another unique observation in IL D39 was that the expression levels of *Scglu* increased significantly in the infected plants (compared with the levels in the mock-treated controls) at the first, third, and fourth time-points.

The *Scglu* and *ScBx4* expression levels similarly increased only in line D39. This should result in an increase in β-glucosidase production and, consequently, enhanced efficiency of glucoside hydrolysis. Contrary to the expectation, the induction of *Scglu* expression was not accompanied by increased aglucone contents at the first and third time-points. At the last time-point, the DIBOA and DIMBOA contents increased admittedly, but not significantly. These findings suggest the *Scglu* expression level is not related to the accumulation of both aglucones, at least at the examined time-points. In lines D33 and L318, the *Scglu* expression level usually decreased, which is consistent with the results of a study by Gomez-Anduro *et al*. [34], in which salt stress and mechanical damage negatively influenced the expression of the maize *glu2* gene.

#### Effect of the *Prs* infection on BX contents

Similar to the gene expression changes, the BX contents decreased (even more frequently) after the BR infection relative to the corresponding levels in the mock-treated plants at four time-points. In contrast to the observed gene expression, there were no clear relationships between the type of reaction and the defence reaction type. Nevertheless, two common features were detected. First, the decreased abundance of DIBOA and DIMBOA was a natural consequence of the downregulated expression of *Scglu*. Interestingly, at the most critical time-point, 24 hpt, the decrease in the DIBOA content was inversely related to the disease resistance of the ILs. Regarding DIMBOA, its content decreased the most in D39, although the decreases in the other two lines were similar. Consistent with our findings, Yang *et al*. [23] reported that in maize, the resistance to northern corn leaf blight caused by *E*. *turcicum* is related to a decrease in the abundance of DIMBOA. In contrast, Ahmad *et al*. [20] reported that *E*. *turcicum* induces the accumulation of apoplastic BXs during the early infection stages, which may inhibit the ability of the fungus to penetrate plant tissues. Similarly, Yu *et al*. [42] speculated that maize plants resist infections by the biotroph *Sporisorium reilianum* f. sp. *zeae* via DIMBOA synthesis and Song *et al*. [21] demonstrated the positive association between DIMBOA accumulation and the resistance to sheath blight disease caused by the necrotrophic fungus *R*. *solani*.

The second common feature was that the *Prs* infection in our study decreased the contents of two glucosides, GDIBOA and GDIMBOA, at a key time-point (24 hpt), during which pathogen growth intensifies and the growing conditions change. Therefore, our results regarding these two glucosides differ completely from those obtained by Yang *et al*. [23], who proved that in addition to DIMBOA contents, the abundance of GDIMBOA (and two other aglucones) is negatively correlated with the resistance to northern corn leaf blight. However, our findings are similar to those reported by Oikawa *et al*. [43], who proved that southern corn leaf blight caused by *Bipolaris maydis*, which has a pathogenesis similar to that of *Prs*, increases the content of another glucoside, 2-hydroxy-4,7-dimethoxy-1,4-benzoxazin-3-one glucoside.

An additional and important question we wanted to answer was whether the stress-induced upregulated and downregulated gene expression levels were accompanied by the same changes to BX contents. In many cases, these processes were coordinated (e.g., downregulated *ScIgl* expression followed by a decrease in the DIMBOA content in L318 at the first time-point; downregulated *ScBx5* expression followed by a decrease in the DIBOA and DIMBOA contents in D33 at 24 hpt; and the upregulated expression of *ScBx1*, *ScBx2*, and *ScBx4* followed by an increase in the GDIMBOA content in line D39 at 8 hpt). However, a lack of correlation was also relatively frequently detected. It is unknown why downregulated *ScBx5* expression is correlated with a decrease in HBOA accumulation. The *ScBx5* gene controls BX biosynthesis downstream of HBOA. One possible explanation for the effects of the changes to *ScBx5* expression may involve an unknown type of feedback. Another unexplained phenomenon was the simultaneous decrease in the DIBOA content and an increase in both glucosides (GDIBOA and GDIMBOA). Specifically, a lack of coordination between gene expression levels and BX contents was apparent in line D39. The HBOA, DIBOA, DIMBOA, and MBOA contents in D39 decreased in response to *Prs*, whereas the expression of the genes controlling their biosynthesis (especially at the first and last time-points) increased. It is also unclear why in line D39 the *Scglu* expression level increased significantly, but the abundance of GDIBOA and GDIMBOA decreased at 8, 24, and 48 hpt. The few published articles relevant to the current study describe how the fungal-induced changes in *Bx* gene expression and BX contents are synchronized. For example, a study by Ding *et al*. [36] examined the activation of BX synthesis and selected defence gene expression after treatments with elicitors from the pepper pathogen *P*. *capsici* and pepper root exudates. The authors proved that in many cases, upregulated or downregulated *Bx1* expression is not accompanied by a similar change to metabolite contents. For example, at 48 h after the treatment with a spore lysis suspension, the *Bx1* expression level decreased, whereas DIBOA and MBOA accumulation increased and the DIMBOA content was unchanged. At 24 h after the treatment with the spore culture suspension, *Bx1* was expressed at lower levels in the infected plants than in the untreated control plants, but the DIBOA and DIMBOA contents exhibited the opposite pattern. Nevertheless, they revealed a strong correlation between *Bx1* expression and the DIMBOA content, which differs considerably from our study. Accordingly, in rye, the changes to gene expression and BX contents appear to occur independently or they are not temporally coordinated. The accumulation of specific transcripts is most probably sufficient for ensuring an increase in metabolite production, specifically GDIBOA and GDIMBOA in plants infected with *Prs* and MBOA in mock-treated plants.

The data presented herein indicate that some BXs (mainly GDIBOA and GDIMBOA) are substantial components of induced rye defences against BR. The significant absolute and relative increases in their contents occur at key moments for disease development, which is when HMCs start to form, pathogen growth is extensive, and necrotic lesions are developing. Although the abundance of three other BXs, namely DIBOA, DIMBOA, which may be involved in maize innate immunity against *Setosphaeria turcica* as extracellular signals for the PAMP-induced callose [20], and MBOA, increases as early as 8 hpt, these BXs are probably not associated with immunity because they are induced by both *Prs* and water. Additionally, the MBOA content steadily increased up to 48 hpt, implying this BX has a substantial role in rye responses to stresses related to the infection procedure. In contrast, HBOA appears to minimally affect the resistance mechanisms mobilized by *Prs*.

In summary, a *Prs* infection and the treatment procedure itself affect both gene expression and BX contents in rye. The key components of the defence response of all analysed rye ILs against BR are as follows: *ScBx1*, *ScBx2*, *ScBx4*, and *Scglu* as well as GDIBOA and GDIMBOA. The changes in the gene expression levels and BX contents are usually positively associated with disease resistance. The intensity of the reaction depends on the genotype, with the most resistant lines mobilizing their defence mechanisms more effectively, in a more coordinated manner, and earlier than the less resistant lines.

Our research is an important step toward characterizing the molecular mechanisms underlying rye defences against BR and for evaluating inoculation procedures. Furthermore, our data may be useful for future transcriptome- and metabolome-based selection of rye germplasm with enhanced resistance to BR. However, there are still many ambiguities that must be clarified, the most important of which is why the BR infection and disease development and the infection procedure cause such different changes to individual metabolites and genes. Future studies should also examine why the treatments with water and *Prs* urediniospores have the opposite effects on gene expression and/or BX contents at the same time-points.

## Material and methods

### Plant materials

The following three rye inbred lines (ILs) were analysed: D33 and D39 (bred by Danko Plant Breeders Ltd., Poland) as well as L318 (bred by the Department of Plant Genetics, Breeding and Biotechnology, Warsaw University of Life Sciences). The ILs were selected based on the BX contents measured after a natural vernalization period. The highest DIBOA content was detected for L318, followed by D33 and then D39. Under field conditions, BR resistance was highest for D33, followed by D39 and then L318 [44; S9 Table].

Plants were cultivated in 24-well trays filled with a mixture of peat and perlite under controlled conditions (22°C with a 16-h light/8-h dark photoperiod). Twelve plants (one replicate) were grown in a single tray segment (7 cm diameter). Experiments were performed with three biological replicates.

### Pathogen

Rye plants were infected with *Prs* single spore isolate No. 1.1.6, which was selected based on a preliminary experiment involving detached-leaf inoculations with 30 isolates. The selected isolate was characterized by the least compatible and most uniform host–pathogen reaction in the tested lines (S10 Table). The detached-leaf test was conducted as described by Hsam *et al*. [45]. For details, please refer to the Supporting Information.

### Establishing the essential time-points for plant–pathogen interactions

The seedlings of susceptible rye cultivar Słowiańskie were inoculated with *Prs* single spore isolate No. 1.1.6. Leaf samples were collected at 4, 8, 12, 16, 20, 24, 36, 48, and 72 hpi and stained with calcofluor white as described by Orczyk *et al*. [46]. Briefly, samples were fixed for 24 h with an ethanol:dichloromethane (3:1) solution supplemented with 0.15% trichloroacetic acid, after which they were rinsed twice with 50% ethanol, twice with 0.05 M sodium hydroxide, three times with water, and once with 0.1 M Tris. They were then stained with calcofluor white (3.5 mg/ml).

The stained leaf fragments were examined with the Diaphot fluorescence microscope (Nikon) for the presence of germinating spores, HMCs, and micronecrosis symptoms. Additionally, the number of infection sites was calculated. Observations were made in 80 on average (but not less than 30) infection sites per leaf sample. For selecting time-points for additional analyses of plant–pathogen interactions, the germinating spores and appressoria at infection sites were counted. The following four infection site profiles were used to reflect plant–pathogen interactions: (i) appressoria; (ii) appressoria and HMCs; (iii) appressoria, HMCs, and micronecrosis; and (iv) appressoria and micronecrosis. The analysis of pathogenesis and the percentage of profiles in the preliminary experiment with cv. Słowiańskie (Fig 1) were calculated according to: Eqs 1, 2, 3 and 4. The analysis of pathogenesis and the percentage of profiles in the main experiment with lines L318, D33 and D39 (Fig 2) were calculated according to: Eqs 5, 6, 7 and 8.

$$\text{Percentage}\;\text{of}\;\text{profile}\;\text{i}=\frac{n_{i}}{\left(n_{gs}+n_{i}+\;n_{ii}+n_{iii}+n_{iv}\right)}\times 100\%$$ (1)

$$\text{Percentage}\;\text{of}\;\text{profile}\;\text{ii}=\frac{n_{ii}}{\left(n_{gs}+n_{i}+n_{ii}+n_{iii}+n_{iv}\right)}\times 100\%$$ (2)

$$\text{Percentage}\;\text{of}\;\text{profile}\;\text{iii}=\frac{n_{iii}}{\left(n_{gs}+n_{i}+n_{ii}+n_{iii}+n_{iv}\right)}\times 100\%$$ (3)

$$\text{Percentage}\;\text{of}\;\text{profile}\;\text{iv}=\frac{n_{iv}}{\left(n_{gs}+n_{i}+n_{ii}+n_{iii}+n_{iv}\right)}\times 100\%$$ (4)

$$\text{Percentage}\;\text{of}\;\text{profile}\;\text{i}=\frac{n_{i}}{\left(n_{i}+n_{ii}+n_{iii}+n_{iv}\right)}\times 100\%$$ (5)

$$\text{Percentage}\;\text{of}\;\text{profile}\;\text{ii}=\frac{n_{ii}}{\left(n_{i}+n_{ii}+n_{iii}+n_{iv}\right)}\times 100\%$$ (6)

$$\text{Percentage}\;\text{of}\;\text{profile}\;\text{iii}=\frac{n_{iii}}{\left(n_{i}+n_{ii}+n_{iii}+n_{iv}\right)}\times 100\%$$ (7)

$$\text{Percentage}\;\text{of}\;\text{profile}\;\text{iv}=\frac{n_{iv}}{\left(n_{i}+n_{ii}+n_{iii}+n_{iv}\right)}\times 100\%$$ (8)

where: n_gs_−number of infection sites with germinating spores, n_i_−number of infection sites with appressoria, n_ii_−number of infection sites with appresoria and HMC, n_iii_−number of infection sites with appresoria, HMC and micronecrosis, n_iv_−number of infection sites with appresoria and micronecrosis.

The number of infection sites with germinating spores and the rates of the four profiles were used to select the following time-points for further analyses of plant–pathogen interactions: 8, 17, 24, and 48 hpi. The first time-point (8 hpi) was selected because of the considerable abundance of appressoria at established infection sites. The 17 and 24 hpi time-points were associated with HMC formation and intense pathogen growth, respectively. The final time-point (48 hpi) corresponded to the beginning of the resistance reaction. Leaf samples were collected at the selected time-points, stained, and analysed regarding plant–pathogen interaction profiles.

### *Prs* and mock treatment

Spores from a single spore isolate of *Prs* were suspended in water with Tween 20 for the subsequent inoculation of three 12-day-old rye IL seedlings. The plants were sprayed with a spore solution or water containing Tween 20 (mock treatment), after which the applied solutions were spread on the leaf surface with a brush. Untreated plants grown under the same conditions as the inoculated and mock-treated plants served as an additional control. Immediately after the inoculation or mock treatment, multiple pots with plants were incubated for 24 h at 18°C in boxes covered with black plastic material to maintain dark and humid conditions. The plants were then transferred to growth chambers (with conditions as described above). The plants were cultivated, inoculated, and histologically analysed as described by [32].

### Sampling of plant materials

At 8, 17, 24, and 48 hpt, the collected plant material for a biological replicate was divided into two equal parts, with one part used for RNA isolation, and the second part analysed for BX content. Additionally, leaf fragments were collected to characterize the infection type. The number of infection sites for each plant–pathogen interaction profile was recorded. Infection types were determined at 15 days post-inoculation (dpi) based on the following 6-point scale [47]: 0 = immune (no visible reaction); 0; = resistant (chlorotic or necrotic flecking) 1 = resistant (minute uredinia surrounded by chlorosis or necrosis); 2 = moderately resistant (small to medium-size uredinia, surrounded by chlorosis or necrosis); 3 = moderately susceptible (medium to large uredinia surrounded by chlorosis); and 4 = susceptible (medium to large uredinia with little or no chlorosis).

The tissues for gene expression and biochemical analyses were frozen. The tissues designated for biochemical analyses were lyophilized (Alpha model 2–4 LDplus; Martin Christ Gefriertrocknungsanlagen GmbH, Osterode am Harz, Germany).

### RNA isolation and reverse transcription for qRT-PCR

The expression levels of the following genes were analysed: *ScBx1–5* (GenBank: KF636825–KF636828 and KF620524), *ScIgl* (GenBank: MN120476), and *Scglu* (GenBank: AY586531.2). Total RNA was extracted from 100 mg untreated, mock-treated, and *Prs*-treated aerial parts of rye plants with the GeneMATRIX Universal RNA Purification Kit (version 1.2) (Eurx, Gdańsk, Poland). The isolated RNA was dissolved in 40 μl RNase-free water, after which the RNA integrity and concentration were measured with the NanoDrop 2000 spectrophotometer. To avoid genomic DNA contamination, the RNA was treated with Turbo DNase (Thermo Fisher Scientific, Waltham, MA, USA). The RNA was then used as the template to synthesize cDNA with the High-Capacity cDNA Reverse Transcription kit (Thermo Fisher Scientific). The resulting cDNA was diluted with RNase-free water.

### Expression analysis of the selected candidate genes

A qRT-PCR assay was completed in a 96-well plate, with three biological and two technical replicates. Each assay included one candidate gene (*ScBx1–5*, *ScIgl*, or *Scglu*) and the *HvAct* reference gene [chosen based on results of the earlier experiments including three reference genes: actin, glyceraldehyde phosphate dehydrogenase (GADPH), and cell division control protein, AAA-superfamily of ATPases]. The qRT-PCR was completed with the LightCycler 96 Real Time System (Roche, Basel, Switzerland), with the following program: 95°C for 600 s; 32 cycles of 95°C for 10 s, 57°C for 10 s, and 72°C for 15 s; 95°C for 10 s, 55°C for 60 s, and 97°C for 1 s. The total volume of the reaction mixture was 20 μl, which contained 4 μl cDNA, 1 μl each gene-specific primer (5 mM), 4 μl RNase-free water, and 10 μl FastStart Essential DNA Green Master (Roche). Candidate gene expression levels were normalized against the *HvAct* expression level according to the 2^−ΔΔCt^ method [48]. Details regarding the gene-specific primers are provided in S11 Table.

### Microscopic analysis

The microscopic analysis of pathogenesis-related processes was performed as described by [32].

### Biochemical analysis

The contents of the following six BXs were assayed as previously described [29,44]. For details, please refer to the Supporting Information.

### Evaluation of the stress effect

To evaluate the effect of the infection by *Prs* urediniospores, the gene expression and BX levels were compared between the inoculated and untreated plants at specific time-points. To dissect the effect of the *Prs* infection, the difference between the gene expression/BX levels of the infected and mock-treated plants was calculated for each time-point. Positive and negative differences were considered to indicate increases and decreases, respectively.

## Supporting information

S1 Table Relative gene expression level of *ScBx1*—*ScBx5*, *ScIgl*, and *Scglu* in untreated seedlings of rye inbred lines, L318, D33, and D39. (DOCX)

S2 Table Relative gene expression level of *ScBx1*—*ScBx5*, *ScIgl*, and *Scglu* in *Prs*- and mock-treated seedlings of rye inbred lines, L318, D33, and D39 at four time-points, 8, 17, 24, and 48 hpt. (DOCX)

S3 Table The differences in gene expression level of *ScBx1*—*ScBx5*, *ScIgl*, and *Scglu* between *Prs*-treated, mock-treated and untreated rye seedlings (dissecting treatment procedure effect). (DOCX)

S4 Table BX synthesis level in untreated seedlings of rye inbred lines, L318, D33, and D39. (DOCX)

S5 Table BX synthesis level in *Prs*- and mock-treated seedlings of rye inbred lines, L318, D33, and D39 at four time-points, 8, 17, 24, and 48 hpt. (DOCX)

S6 Table The differences in BX synthesis level between *Prs*-treated, mock-treated and untreated rye seedlings (dissecting treatment procedure effect). (DOCX)

S7 Table The differences in gene expression level of *ScBx1*—*ScBx5*, *ScIgl*, and *Scglu* between *Prs*- and mock-treated rye seedlings (dissecting brown rust effect). (DOCX)

S8 Table The differences in BX synthesis level between *Prs*- and mock-treated rye seedlings (dissecting brown rust effect). (DOCX)

S9 Table Characteristics of rye inbred lines, L318, D33, and D39, chosen for experiments. (DOCX)

S10 Table Resistance reaction of three rye inbred lines, L318, D33, and D39, determined based on detached-leaf test. (DOCX)

S11 Table Primers used in qRT-PCR reaction. (DOCX)

S1 File (DOCX)
