# Supplementary material for: Changes in benzoxazinoid contents and the expression of the associated genes in rye (Secale cereale L.) due to brown rust and the inoculation procedure
Source: PLoS One. 2020 May 29;15(5):e0233807. doi: 10.1371/journal.pone.0233807 (PMC7259783; doi:10.1371/journal.pone.0233807)
Supplement: S1 Table — (DOCX) [file pone.0233807.s001.docx]

**S1 Table.** **Relative gene expression level of *ScBx1*—*ScBx5*, *ScIgl*, and *Scglu* in untreated seedlings of rye inbred lines, L318, D33, and D39.**

| Inbred line | Relative gene expression level | | | | | | |
| --- | --- | --- | --- | --- | --- | --- | --- |
|  | *ScBx1* | *ScBx2* | *ScBx3* | *ScBx4* | *ScBx5* | *ScIgl* | *Scglu* |
| L318 | 0.2841 | 0.3856 | 1.9735 | 0.9935 | 1.8907 | 0.0023 | 0.2753 |
| D33 | 1.4555 | 1.1078 | 3.6075 | 1.1722 | 1.6602 | 0.0677 | 4.2600 |
| D39 | 0.2683 | 0.5124 | 1.6497 | 0.6406 | 0.5541 | 0.1023 | 0.3655 |
