# Supplementary material for: Changes in benzoxazinoid contents and the expression of the associated genes in rye (Secale cereale L.) due to brown rust and the inoculation procedure
Source: PLoS One. 2020 May 29;15(5):e0233807. doi: 10.1371/journal.pone.0233807 (PMC7259783; doi:10.1371/journal.pone.0233807)
Supplement: S2 Table — (DOCX) [file pone.0233807.s002.docx]

**S2 Table.** **Relative gene expression level of *ScBx1*—*ScBx5*, *ScIgl*, and *Scglu* in *Prs*- and mock-treated seedlings of rye inbred lines, L318, D33, and D39 at four time-points, 8, 17, 24, and 48 hpt.**

| Inbred line | Time point [hpt] | Relative gene expression level | | | | | | | | | | | | | |
| --- | --- | --- | --- | --- | --- | --- | --- | --- | --- | --- | --- | --- | --- | --- | --- |
|  |  | *ScBx1* | | *ScBx2* | | *ScBx3* | | *ScBx4* | | *ScBx5* | | *ScIgl* | | *Scglu* | |
|  |  | *Prs* treated | mock treated | *Prs* treated | mock treated | *Prs* treated | mock treated | *Prs* treated | mock treated | *Prs* treated | mock treated | *Prs* treated | mock treated | *Prs* treated | mock treated |
| L318 | 8 | 0.0026 | 0.0015 | 0.0278 | 0.0216 | 0.0147 | 0.0112 | 0.0246 | 0.0224 | 0.0024 | 0.0023 | 0.0010 | 0.0027 | 0.3662 | 0.3848 |
|  | 17 | 0.0006 | 0.0013 | 0.0176 | 0.0243 | 0.0023 | 0.0046 | 0.0070 | 0.0145 | 0.0015 | 0.0026 | 0.0007 | 0.0008 | 0.1176 | 0.2349 |
|  | 24 | 0.0000 | 0.0004 | 0.0054 | 0.0167 | 0.0010 | 0.0010 | 0.0031 | 0.0097 | 0.0004 | 0.0005 | 0.0003 | 0.0002 | 0.1253 | 0.2200 |
|  | 48 | 0.0001 | 0.0007 | 0.0046 | 0.0062 | 0.2984 | 0.2601 | 0.0948 | 0.0945 | 0.2728 | 0.3027 | 0.0123 | 0.0130 | 0.2266 | 0.2441 |
| D33 | 8 | 0.0060 | 0.0000 | 0.0350 | 0.0036 | 0.0265 | 0.0472 | 0.0172 | 0.0026 | 0.0039 | 0.0047 | 0.0637 | 0.3825 | 0.2246 | 0.0441 |
|  | 17 | 0.0003 | 0.0035 | 0.0069 | 0.0160 | 0.0067 | 0.0206 | 0.0015 | 0.0114 | 0.0021 | 0.0085 | 0.0227 | 0.0184 | 0.0421 | 0.3038 |
|  | 24 | 0.0003 | 0.0004 | 0.0113 | 0.0066 | 0.0054 | 0.0000 | 0.0108 | 0.0040 | 0.0012 | 0.0036 | 0.0063 | 0.0115 | 0.0862 | 0.1124 |
|  | 48 | 0.0009 | 0.0013 | 0.0055 | 0.0029 | 0.3652 | 0.6611 | 0.0863 | 0.0972 | 0.2229 | 0.2614 | 0.1650 | 0.2783 | 0.0504 | 0.0387 |
| D39 | 8 | 0.0156 | 0.0016 | 0.0485 | 0.0081 | 0.0407 | 0.0497 | 0.0298 | 0.0031 | 0.0048 | 0.0039 | 0.0265 | 0.2355 | 0.3604 | 0.0685 |
|  | 17 | 0.0008 | 0.0010 | 0.0090 | 0.0097 | 0.0112 | 0.0191 | 0.0038 | 0.0026 | 0.0015 | 0.0079 | 0.0084 | 0.0009 | 0.0820 | 0.4310 |
|  | 24 | 0.0005 | 0.0000 | 0.0028 | 0.0015 | 0.0136 | 0.0222 | 0.0012 | 0.0003 | 0.0006 | 0.0033 | 0.0087 | 0.0179 | 0.0879 | 0.0129 |
|  | 48 | 0.0023 | 0.0011 | 0.0109 | 0.0024 | 0.2531 | 0.5327 | 0.0633 | 0.0589 | 0.0495 | 0.1147 | 0.0321 | 0.2031 | 3.0902 | 0.4555 |
