# Supplementary material for: Changes in benzoxazinoid contents and the expression of the associated genes in rye (Secale cereale L.) due to brown rust and the inoculation procedure
Source: PLoS One. 2020 May 29;15(5):e0233807. doi: 10.1371/journal.pone.0233807 (PMC7259783; doi:10.1371/journal.pone.0233807)
Supplement: S3 Table — (DOCX) [file pone.0233807.s003.docx]

**S3 Table. The differences in gene expression level of *ScBx1*—*ScBx5*, *ScIgl*, and *Scglu* between *Prs*-treated, mock-treated and untreated rye seedlings (dissecting treatment procedure effect).**

| Inbred line | Time point  [hpt] | Gene expression level | | | | | | | | | | | | | |
| --- | --- | --- | --- | --- | --- | --- | --- | --- | --- | --- | --- | --- | --- | --- | --- |
|  |  | *ScBx1* | | *ScBx2* | | *ScBx3* | | *ScBx4* | | *ScBx5* | | *ScIgl* | | *Scglu* | |
|  |  | *Prs*-0 | mock-0 | *Prs*-0 | mock-0 | *Prs*-0 | mock-0 | *Prs*-0 | mock-0 | *Prs*-0 | mock-0 | *Prs*-0 | mock-0 | *Prs*-0 | mock-0 |
| L318 | 8 | -0.2815^*a^ | -0.2826^*a^ | -0.3578^*a^ | -0.3640^*a^ | -1.9588^*a^ | -1.9623^*a^ | -0.9689^*a^ | -0.9711^*a^ | -1.8883^*b^ | -1.8884^*b^ | -0.0013^*b^ | 0.0004 ^b^ | 0.0909 ^a^ | 0.1095 ^a^ |
|  | 17 hpi | -0.2835^*a^ | -0.2828^*a^ | -0.3680^*a^ | -0.3613^*a^ | -1.9712^*a^ | -1.9689^*a^ | -0.9865^*a^ | -0.9823^*a^ | -1.8892^*b^ | -1.8881^*b^ | -0.0016^*b^ | -0.0015^*b^ | -0.1577 ^b^ | -0.0404 ^a^ |
|  | 24 hpi | -0.2841^*a^ | -0.2837^*a^ | -0.3802^*a^ | -0.3689^*a^ | -1.9725^*a^ | -1.9725^*a^ | -0.9904^*a^ | -0.9838^*a^ | -1.8903^*b^ | -1.8902^*b^ | -0.0020^*b^ | -0.0021^*b^ | -0.1500^*b^ | -0.0553 ^a^ |
|  | 48 hpi | -0.2840^*a^ | -0.2834^*a^ | -0.3809^*a^ | -0.3793^*a^ | -1.6751^*a^ | -1.7134^*a^ | -0.8987^*a^ | -0.8990^*a^ | -1.6179^*a^ | -1.5880^*a^ | 0.0100^*a^ | 0.0107^*a^ | -0.0487 ^ab^ | -0.0312 ^a^ |
| D33 | 8 | -1.4495^*a^ | -1.4555^*a^ | -1.0728^*a^ | -1.1042^*a^ | -3.5810^*b^ | -3.5603^*b^ | -1.1550^*b^ | -1.1696^*b^ | -1.6563^*b^ | -1.6555^*b^ | -0.0040 ^b^ | 0.3148^*a^ | -4.0354^*a^ | -4.2159^*a^ |
|  | 17 hpi | -1.4552^*a^ | -1.4520^*a^ | -1.1009^*a^ | -1.0918^*a^ | -3.6008^*b^ | -3.5869^*b^ | -1.1707^*b^ | -1.1608^*b^ | -1.6581^*b^ | -1.6517^*b^ | -0.0450^*c^ | -0.0493^*c^ | -4.2179^*a^ | -3.9562^*a^ |
|  | 24 hpi | -1.4552^*a^ | -1.4551^*a^ | -1.0965^*a^ | -1.1012^*a^ | -3.6021^*b^ | -3.6075^*b^ | -1.1614^*b^ | -1.1682^*b^ | -1.6590^*b^ | -1.6566^*b^ | -0.0614^*c^ | -0.0562^*c^ | -4.1738^*a^ | -4.1476^*a^ |
|  | 48 hpi | -1.4546^*a^ | -1.4542^*a^ | -1.1023^*a^ | -1.1049^*a^ | -3.2423^*a^ | -2.9464^*a^ | -1.0859^*a^ | -1.0750^*a^ | -1.4373^*a^ | -1.3988^*a^ | 0.0973^*a^ | 0.2106^*b^ | -4.2096^*a^ | -4.2213^*a^ |
| D39 | 8 | -0.2527^*a^ | -0.2667^*a^ | -0.4639^*a^ | -0.5043^*a^ | -1.6090^*a^ | -1.6000^*b^ | -0.6108^*a^ | -0.6375^*a^ | -0.5493^*a^ | -0.5502^*a^ | -0.0758 ^a^ | 0.1332 ^a^ | -0.0051 ^b^ | -0.2970^*ab^ |
|  | 17 hpi | -0.2675^*a^ | -0.2673^*b^ | -0.5034^*b^ | -0.5027^*a^ | -1.6385^*a^ | -1.6306^*b^ | -0.6368^*a^ | -0.6380^*a^ | -0.5526^*a^ | -0.5462^*a^ | -0.0939^*a^ | -0.1014^*b^ | -0.2835^*b^ | 0.0655 ^a^ |
|  | 24 hpi | -0.2678^*a^ | -0.2683^*b^ | -0.5096^*b^ | -0.5109^*a^ | -1.6361^*a^ | -1.6275^*b^ | -0.6394^*a^ | -0.6403^*a^ | -0.5535^*a^ | -0.5508^*a^ | -0.0936^*a^ | -0.0844^*ab^ | -0.2776^*b^ | -0.3526^*b^ |
|  | 48 hpi | -0.2660^*a^ | -0.2672^*b^ | -0.5015^*b^ | -0.5100^*a^ | -1.3966^*a^ | -1.1170^*a^ | -0.5773^*a^ | -0.5817^*a^ | -0.5046^*a^ | -0.4394^*a^ | -0.0702 ^a^ | 0.1008 ^ab^ | 2.7247^*a^ | 0.0900 ^a^ |

*) differences between the values of gene expression level measured in infected with *Prs* or mock-treated and untreated seedlings, “*Prs*-0” and “mock-0”, respectively; statistically significant at p < 0.05 (based on Mann-Whitney U test); the homogenous groups within *Prs*- or mock-treated plants, determined on the basis of Fisher's least significant difference procedure are marked with the same letter

underlined – statistically significant differences between *Prs*- and mock-treated plants at a given time-point
