# Supplementary material for: Changes in benzoxazinoid contents and the expression of the associated genes in rye (Secale cereale L.) due to brown rust and the inoculation procedure
Source: PLoS One. 2020 May 29;15(5):e0233807. doi: 10.1371/journal.pone.0233807 (PMC7259783; doi:10.1371/journal.pone.0233807)
Supplement: S4 Table — (DOCX) [file pone.0233807.s004.docx]

**S4 Table.** **BX synthesis level in untreated seedlings of rye inbred lines, L318, D33, and D39.**

| Inbred line | HBOA | GDIBOA | DIBOA | GDIMBOA | DIMBOA | MBOA |
| --- | --- | --- | --- | --- | --- | --- |
| L318 | 0.2159 | 2.6270 | 4.7522 | 0.2430 | 0.0143 | 0.0480 |
| D33 | 0.2033 | 2.5656 | 8.3730 | 0.1084 | 0.0470 | 0.0091 |
| D39 | 0.1281 | 3.3547 | 4.7559 | 0.0739 | 0.0021 | 0.0059 |
