# Supplementary material for: Changes in benzoxazinoid contents and the expression of the associated genes in rye (Secale cereale L.) due to brown rust and the inoculation procedure
Source: PLoS One. 2020 May 29;15(5):e0233807. doi: 10.1371/journal.pone.0233807 (PMC7259783; doi:10.1371/journal.pone.0233807)
Supplement: S5 Table — (DOCX) [file pone.0233807.s005.docx]

**S5 Table.** **BX synthesis level in *Prs*- and mock-treated seedlings of rye inbred lines, L318, D33, and D39 at four time-points, 8, 17, 24, and 48 hpt.**

| Inbred line | Time point [hpt] | BX content [µg/mg d.m.] | | | | | | | | | | | |
| --- | --- | --- | --- | --- | --- | --- | --- | --- | --- | --- | --- | --- | --- |
|  |  | HBOA | | GDIBOA | | DIBOA | | GDIMBOA | | DIMBOA | | MBOA | |
|  |  | *Prs* | mock | *Prs* | mock | *Prs* | mock | *Prs* | mock | *Prs* | mock | *Prs* | mock |
| L318 | 8 | 0,0276 | 0,0572 | 4,5352 | 3,6668 | 4,0120 | 6,2596 | 0,5148 | 0,4272 | 0,0517 | 0,1032 | 0,9854 | 3,1050 |
|  | 17 | 0.0194 | 0.0425 | 4.6405 | 2.7428 | 2.1995 | 5.0461 | 0.5291 | 0.4359 | 0.0299 | 0.0982 | 0.6450 | 2.3684 |
|  | 24 | 0.0145 | 0.0467 | 4.8855 | 3.9160 | 1.6274 | 4.8787 | 0.7200 | 0.4023 | 0.0253 | 0.0729 | 0.5640 | 2.1789 |
|  | 48 | 0.0077 | 0.0371 | 3.8428 | 3.8553 | 0.5928 | 3.1234 | 0.5506 | 0.5445 | 0.0126 | 0.0551 | 0.1692 | 1.3466 |
| D33 | 8 | 0.0263 | 0.0583 | 5.3205 | 5.6933 | 3.2004 | 6.6042 | 0.3908 | 0.3008 | 0.0238 | 0.0432 | 0.4536 | 1.4423 |
|  | 17 | 0.0201 | 0.0274 | 4.8322 | 5.8305 | 2.4078 | 3.8479 | 0.4766 | 0.5771 | 0.0250 | 0.0347 | 0.8066 | 1.1060 |
|  | 24 | 0.0079 | 0.0308 | 4.9963 | 4.3648 | 0.8594 | 3.8503 | 0.5655 | 0.4469 | 0.0078 | 0.0390 | 0.3990 | 1.3916 |
|  | 48 | 0.0111 | 0.0331 | 4.3644 | 4.1328 | 1.3177 | 4.7469 | 0.4529 | 0.3415 | 0.0164 | 0.0469 | 0.6758 | 0.5697 |
| D39 | 8 | 0.0147 | 0.0465 | 5.8705 | 4.4893 | 1.6331 | 5.7195 | 0.6992 | 0.4252 | 0.0197 | 0.0979 | 0.6044 | 2.1756 |
|  | 17 | 0.0116 | 0.0492 | 5.1356 | 4.2196 | 1.9793 | 6.5725 | 0.7313 | 0.4529 | 0.0210 | 0.1034 | 0.6366 | 1.9263 |
|  | 24 | 0.0073 | 0.0236 | 4.3583 | 3.2844 | 0.8114 | 3.2781 | 0.6520 | 0.3305 | 0.0104 | 0.0435 | 0.6409 | 1.1280 |
|  | 48 | 0.0146 | 0.0147 | 3.4786 | 4.4970 | 2.3911 | 1.4348 | 0.3513 | 0.5183 | 0.0298 | 0.0194 | 0.4856 | 0.7182 |
