# Supplementary material for: Changes in benzoxazinoid contents and the expression of the associated genes in rye (Secale cereale L.) due to brown rust and the inoculation procedure
Source: PLoS One. 2020 May 29;15(5):e0233807. doi: 10.1371/journal.pone.0233807 (PMC7259783; doi:10.1371/journal.pone.0233807)
Supplement: S6 Table — (DOCX) [file pone.0233807.s006.docx]

**S6 Table.** **The differences in BX synthesis level between *Prs*-treated, mock-treated and untreated rye seedlings (dissecting treatment procedure effect).**

| Inbred line | Time point [hpt] | BX content [µg/mg d.m.] | | | | | | | | | | | |
| --- | --- | --- | --- | --- | --- | --- | --- | --- | --- | --- | --- | --- | --- |
|  |  | HBOA | | GDIBOA | | DIBOA | | GDIMBOA | | DIMBOA | | MBOA | |
|  |  | *Prs*-0 | mock-0 | *Prs*-0 | mock-0 | *Prs*-0 | mock-0 | *Prs*-0 | mock-0 | *Prs*-0 | mock-0 | *Prs*-0 | mock-0 |
| L318 | 8 | -0.1883^*a^ | -0.1587^*a^ | 1.9082^*a^ | 1.0398^*a^ | -0.7402^*a^ | 1.5074^*a^ | 0.2718^*a^ | 0.1842^*a^ | 0.0374^*a^ | 0.0889^*a^ | 0.9711^*a^ | 3.0907^*a^ |
|  | 17 | -0.1965^*a^ | -0.1734^*a^ | 2.0135^*a^ | 0.1158^*a^ | -2.5527^*ab^ | 0.2939^*a^ | 0.2861^*ab^ | 0.1929^*a^ | 0.0156^*ab^ | 0.0839^*a^ | 0.6307^*a^ | 2.3541^*ab^ |
|  | 24 | -0.2014^*a^ | -0.1692^*a^ | 2.2585^*a^ | 1.2890^*a^ | -3.1248^*b^ | 0.1265^*a^ | 0.4770^*ab^ | 0.1593^*a^ | 0.0110^*b^ | 0.0586^*ab^ | 0.5497^*ab^ | 2.1646^*ab^ |
|  | 48 | -0.2082^*a^ | -0.1788^*a^ | 1.2158^*a^ | 1.2283^*a^ | -4.1594^*b^ | -1.6288^*a^ | 0.3076^*b^ | 0.3015^*a^ | -0.0017^*b^ | 0.0408^*b^ | 0.1549^*b^ | 1.3323^*b^ |
| D33 | 8 | -0.1770^*a^ | -0.1450^*a^ | 2.7549^*a^ | 3.1277^*a^ | -5.1726^*a^ | -1.7688^*a^ | 0.2824^*b^ | 0.1924^*b^ | -0.0232^*a^ | -0.0038^*a^ | 0.4445^*b^ | 1.4332^*a^ |
|  | 17 | -0.1832^*a^ | -0.1759^*a^ | 2.2666^*ab^ | 3.2649^*b^ | -5.9652^*ab^ | -4.5251^*b^ | 0.3682^*ab^ | 0.4687^*a^ | -0.0220^*a^ | -0.0123^*a^ | 0.7975^*a^ | 1.0969^*ab^ |
|  | 24 | -0.1954^*a^ | -0.1725^*a^ | 2.4307^*ab^ | 1.7992^*b^ | -7.5136^*c^ | -4.5227^*b^ | 0.4571^*a^ | 0.3385^*ab^ | -0.0392^*a^ | -0.0080^*a^ | 0.3899^*b^ | 1.3825^*a^ |
|  | 48 | -0.1922^*a^ | -0.1702^*a^ | 1.7988^*b^ | 1.5672^*b^ | -7.0553^*bc^ | -3.6261^*ab^ | 0.3445^*b^ | 0.2331^*b^ | -0.0306^*a^ | -0.0001^*a^ | 0.6667^*ab^ | 0.5606^*b^ |
| D39 | 8 | -0.1134^*a^ | -0.0816^*a^ | 2.5158^*a^ | 1.1346^*a^ | -3.1228^*ab^ | 0.9636^*ab^ | 0.6253^*a^ | 0.3513^*ab^ | 0.0176^*ab^ | 0.0958^*a^ | 0.5985^*a^ | 2.1697^*a^ |
|  | 17 | -0.1165^*a^ | -0.0789^*a^ | 1.7809^*b^ | 0.8649^*a^ | -2.7766^*a^ | 1.8166^*a^ | 0.6574^*a^ | 0.3790^*ab^ | 0.0189^*a^ | 0.1013^*a^ | 0.6307^*a^ | 1.9204^*a^ |
|  | 24 | -0.1208^*a^ | -0.1045^*a^ | 1.0036^*c^ | -0.0703^*a^ | -3.9445^*b^ | -1.4778^*bc^ | 0.5781^*a^ | 0.2566^*b^ | 0.0083^*b^ | 0.0414^*b^ | 0.6350^*a^ | 1.1221^*ab^ |
|  | 48 | -0.1135^*a^ | -0.1134^*a^ | 0.1239^*d^ | 1.1423^*a^ | -2.3648^*a^ | -3.3211^*c^ | 0.2774^*b^ | 0.4444^*a^ | 0.0277^*a^ | 0.0173^*b^ | 0.4797^*a^ | 0.7123^*b^ |

*) differences between the values of BX synthesis level measured in infected with *Prs* or mock-treated and untreated seedlings, “*Prs*-0” and “mock-0”, respectively; statistically significant at p < 0.05 (based on Mann-Whitney U test); the homogenous groups within *Prs*- or mock-treated plants, determined on the basis of Fisher's least significant difference procedure are marked with the same letter

underlined – statistically significant differences between *Prs*- and mock-treated plants at a given time point
