# Supplementary material for: Changes in benzoxazinoid contents and the expression of the associated genes in rye (Secale cereale L.) due to brown rust and the inoculation procedure
Source: PLoS One. 2020 May 29;15(5):e0233807. doi: 10.1371/journal.pone.0233807 (PMC7259783; doi:10.1371/journal.pone.0233807)
Supplement: S7 Table — (DOCX) [file pone.0233807.s007.docx]

**S7 Table. The differences in gene expression level of *ScBx1*—*ScBx5*, *ScIgl*, and *Scglu* between *Prs*- and mock-treated rye seedlings (dissecting brown rust effect).**

| Inbred line | Time point [hpt] | Gene expression level | | | | | | |
| --- | --- | --- | --- | --- | --- | --- | --- | --- |
|  |  | *ScBx1* | *ScBx2* | *ScBx3* | *ScBx4* | *ScBx5* | *ScIgl* | *Scglu* |
| L318 | 8 | 0.0011 | 0.0063 | 0.0036 | 0.0022 | 0.0001 | -0.0016* | -0.0186 |
|  | 17 | -0.0007 | -0.0067 | -0.0024 | -0.0075 | -0.0011* | -0.0001 | -0.1173 |
|  | 24 | -0.0004* | -0.0113* | 0.0000 | -0.0066* | -0.0001 | 0.0000 | -0.0947 |
|  | 48 | -0.0006* | -0.0016 | 0.0383 | 0.0003 | -0.0299 | -0.0007 | -0.0175 |
| D33 | 8 | 0.0060* | 0.0314 | -0.0207 | 0.0146 | -0.0008 | -0.3188* | 0.1806 |
|  | 17 | -0.0033* | -0.0091* | -0.0140* | -0.0099* | -0.0064* | 0.0043 | -0.2618* |
|  | 24 | 0.0000 | 0.0047 | 0.0054 | 0.0068 | -0.0024* | -0.0051* | -0.0262 |
|  | 48 | -0.0003 | 0.0026* | -0.2959* | -0.0109 | -0.0385 | -0.1133* | 0.0116 |
| D39 | 8 | 0.0140* | 0.0404* | -0.0090 | 0.0267* | 0.0009 | -0.2090 | 0.2919* |
|  | 17 | -0.0002 | -0.0007 | -0.0079* | 0.0012 | -0.0064* | 0.0075* | -0.3490* |
|  | 24 | 0.0005 | 0.0013 | -0.0085* | 0.0009 | -0.0028* | -0.0093 | 0.0750* |
|  | 48 | 0.0013* | 0.0086* | -0.2796* | 0.0045 | -0.0652* | -0.1710 | 2.6347* |

*) differences between the values of gene expression level measured in infected with *Prs* and mock-treated seedlings statistically significant at p < 0.05 (based on Mann-Whitney U test)
