# Supplementary material for: Changes in benzoxazinoid contents and the expression of the associated genes in rye (Secale cereale L.) due to brown rust and the inoculation procedure
Source: PLoS One. 2020 May 29;15(5):e0233807. doi: 10.1371/journal.pone.0233807 (PMC7259783; doi:10.1371/journal.pone.0233807)
Supplement: S8 Table — (DOCX) [file pone.0233807.s008.docx]

**S8 Table. The differences in BX synthesis level between *Prs*- and mock-treated rye seedlings (dissecting brown rust effect).**

| Inbred line | Time point [hpt] | BX content [µg/mg d.m.] | | | | | |
| --- | --- | --- | --- | --- | --- | --- | --- |
|  |  | HBOA | GDIBOA | DIBOA | GDIMBOA | DIMBOA | MBOA |
| L318 | 8 | -0.0296 | 0.8684 | -2.2476 | 0.0876 | -0.0515* | -2.1196* |
|  | 17 | -0.0231* | 1.8977* | -2.8466* | 0.0933 | -0.0683* | -1.7235* |
|  | 24 | -0.0322* | 0.9695 | -3.2513* | 0.3176* | -0.0476* | -1.6149* |
|  | 48 | -0.0295* | -0.0125 | -2.5306* | 0.0061 | -0.0425* | -1.1774* |
| D33 | 8 | -0.0319* | -0.3728 | -3.4038 | 0.0900 | -0.0194 | -0.9887* |
|  | 17 | -0.0072 | -0.9983* | -1.4401* | -0.1005 | -0.0096 | -0.2994 |
|  | 24 | -0.0228* | 0.6315 | -2.9909* | 0.1186 | -0.0312* | -0.9925 |
|  | 48 | -0.0220* | 0.2316 | -3.4292* | 0.1114 | -0.0306* | 0.1061 |
| D39 | 8 | -0.0318* | 1.3812 | -4.0864* | 0.2740* | -0.0782* | -1.5712* |
|  | 17 | -0.0376* | 0.9159* | -4.5932* | 0.2784* | -0.0824* | -1.2897* |
|  | 24 | -0.0162* | 1.0739* | -2.4667* | 0.3214* | -0.0330* | -0.4871 |
|  | 48 | -0.0001 | -1.0184 | 0.9563 | -0.1670* | 0.0104 | -0.2326 |

*) differences between the values of BX synthesis level measured in infected with *Prs* and mock-treated seedlings statistically significant at p < 0.05 (based on Mann-Whitney U test)
