# Supplementary material for: Changes in benzoxazinoid contents and the expression of the associated genes in rye (Secale cereale L.) due to brown rust and the inoculation procedure
Source: PLoS One. 2020 May 29;15(5):e0233807. doi: 10.1371/journal.pone.0233807 (PMC7259783; doi:10.1371/journal.pone.0233807)
Supplement: S9 Table — (DOCX) [file pone.0233807.s009.docx]

**Table S9** Characteristics of rye inbred lines, L318, D33, and D39, chosen for experiments^*)^.

|  | Content^*)^ [µg/mg d.m.] of BXs in aerial parts of plants grown under field conditions | | | | | | | Resistance to brown rust^**)^ evaluated in field conditions^*)^ |
| --- | --- | --- | --- | --- | --- | --- | --- | --- |
| Inbred line | HBOA | GDIBOA | DIBOA | GDIMBOA | DIMBOA | MBOA | sum |  |
| L318 | 0.0130 | 0.0259 | 1.1788 | 0.0000 | 0.0000 | 0.0000 | 1.2177 | 4.0 |
| D33 | 0.0153 | 0.1736 | 0.8459 | 0.0019 | 0.0000 | 0.0015 | 1.0382 | 2.4 |
| D39 | 0.0099 | 0.1755 | 0.4760 | 0.0003 | 0.0000 | 0.0012 | 0.6639 | 3.3 |

^*)^ mean values from two seasons (2014, 2015) and two locations (Danko Hodowla Roślin Ltd., and West Pomeranian University of Technology Szczecin)

^**)^ plant infection level was evaluated according to a six-degree scale:

0 – resistant, no symptoms on plants

1 – resistant, chlorosis/necrosis and/or single uredinia sporadically visible on leaves

2 – moderate resistant; single small uredinia regularly present on majority of plants

3 – moderately susceptible; up to 40% of leaf surface covered with uredinia

4 – susceptible; up to 70% of leaf surface covered with uredinia

5 – very susceptible; over 70% of leaf surface covered with uredinia.

^*)^ based on results of association analysis of single nucleotide polymorphisms associated with brown rust resistance published in Rakoczy-Trojanowska et al;. 2017. DOI 10.1007/s11105-017-1030-6
