# Supplementary material for: Changes in benzoxazinoid contents and the expression of the associated genes in rye (Secale cereale L.) due to brown rust and the inoculation procedure
Source: PLoS One. 2020 May 29;15(5):e0233807. doi: 10.1371/journal.pone.0233807 (PMC7259783; doi:10.1371/journal.pone.0233807)
Supplement: S10 Table — (DOCX) [file pone.0233807.s010.docx]

**S10 Table. Resistance reaction of three rye inbred lines, L318, D33, and D39, determined based on detached-leaf test.**

| Inbred line |  | Isolate | | | | | | | | | | | | | | | | | | | | | | | | |  | | | |
| --- | --- | --- | --- | --- | --- | --- | --- | --- | --- | --- | --- | --- | --- | --- | --- | --- | --- | --- | --- | --- | --- | --- | --- | --- | --- | --- | --- | --- | --- | --- |
|  | **1.1.6** | 6 | 16 | 20.1 | 27.2 | 28.1.5 | 34.1.5 | 38.1.8 | 45.1.7 | 47.1 | 49 | 49.2.5 | 49.2.6 | 50 | 52.1 | 52.7 | 57 | 60.2 | 62.2 | 63.1 | 67 | 68.2.2 | 69.2.2 | 71.1 | 72.2 | 75.6 | 76.2 | 76.2.1.1 | 76.2.1.2 | 88.2.9.3 |
| D39 | **0;-1** | 2-3 | 0-1 | 2-3 | 2-3 | 2-3 | 2-3 | 1-2 | 2 | 3-4 | 3-4 | 3-4 | 2-3 | 0-1 | 2-3 | 3-4 | 2-3 | 3-4 | 3-4 | 3-4 | 3-4 | 3-4 | 2 | 3-4 | 2-3 | 4 | 3-4 | 0 | 0 | 0; |
| D33 | **0-0;** | 0; | 2-3 | 2-3 | 1-2 | 2-3 | 0 | 0 | 1-2 | 0; | 4 | 1-2 | 2-3 | 4 | 1-2 | 3-4 | 2-3 | 0; | 0;-1 | 1-2 | 0;-1 | 0; | 3-4 | 2-3 | 2-3 | 0; | 4 | 0-1 | 1-2 | 2-3 |
| L318 | **2-3** | 3-4 | 2 | 2-3 | 1-2 | 3 | 2-3 | 2-3 | 1-2 | 2-3 | 3-4 | 3-4 | 2-3 | 3 | 3 | 2-3 | 3 | 3-4 | 2-3 | 0-3 | 4 | 4 | 3-4 | 2-3 | 2-3 | 4 | 4 | 2-3 | 1-2 | 3-4 |

infection types according to Murphy (1935)

isolate selected for the main experiment is marked in bold
