# Supplementary material for: Changes in benzoxazinoid contents and the expression of the associated genes in rye (Secale cereale L.) due to brown rust and the inoculation procedure
Source: PLoS One. 2020 May 29;15(5):e0233807. doi: 10.1371/journal.pone.0233807 (PMC7259783; doi:10.1371/journal.pone.0233807)
Supplement: S11 Table — (DOCX) [file pone.0233807.s011.docx]

**S11 Table. Primers used in qRT-PCR reaction.**

| Gene | Sequences (5’ – 3’) |
| --- | --- |
| *ScBx1* | F: TCAAAACCTGAACACGTGAAGC |
|  | R: GCCTCTAGCCTTTTCAATCCTTC |
| *ScBx2* | F: CTCATGATTCCACACTTCTCCC |
|  | R: AGGCGTTTACAACGACACGA |
| *ScBx3* | F: CGGTCTCACTACGGATAACATCA |
|  | R: GAGCTCAGCCATGCCG |
| *ScBx4* | F: TTCTCTCAAGAAAGAGTACGGC |
|  | R: GGAGTATTCCAGCACCAGGA |
| *ScBx5* | F: GAAGCTCGTCAACACCCATCT |
|  | R: GCCAGGAACTCGCTCATGT |
| *ScIgl* | F: AACACCAGCTACACCATCAGAG |
|  | R: GTGGGTTTACAGTCGCCCTA |
| *Scglu* | F: CAATTTGGCACTGGGACACC |
|  | R: CACAGCTCGGCGAAGTATTTG |
| *HvAct* | F: CCCCTTTGAACCCAAAAGCC |
|  | R: GAAAGCACGGCCTGAATAGC |
