# Supplementary material for: Changes in benzoxazinoid contents and the expression of the associated genes in rye (Secale cereale L.) due to brown rust and the inoculation procedure
Source: PLoS One. 2020 May 29;15(5):e0233807. doi: 10.1371/journal.pone.0233807 (PMC7259783; doi:10.1371/journal.pone.0233807)
Supplement: S1 File — (DOCX) [file pone.0233807.s012.docx]

**Supporting information – materials and methods**

1. **Details of detached leaf test**

The leaf segments 2–3 cm from 10-day-old plants (10 plants per line) were added to Petri dishes filled with 0.6% agar containing 3,4 mM benzimidazole. A spore settling tower was used to inoculate leaves with 500–700 *Prs* urediniospores/cm2. The infected samples were incubated in a phytotron for 10 days under the following conditions: 18 °C, 70% relative humidity, 4 klx light intensity, and a 16-h light/8-h dark photoperiod. The reaction of each line to the infection was evaluated based on the following scale, which was slightly modified from that described by Murphy (1935): 0 = immune (no visible reaction); 0; = highly resistant (chlorotic or necrotic flecking); 1 = resistant (minute uredinia surrounded by chlorosis or necrosis); 2 = moderately resistant (small pustules surrounded by chlorosis); 3 = moderately susceptible (moderately large pustules surrounded by chlorosis); and 4 = susceptible (large to moderately large pustules with little or no chlorosis).

1. **Details of biochemical analysis**

HBOA, GDIBOA, DIBOA, GDIMBOA, DIMBOA, and MBOA. Briefly, plant samples were treated with 70% methanol containing an internal standard (2 µg/ml indoxyl β-D-glucoside) at 10 MPa operating pressure and 40 °C with the ASE-200 accelerated solvent extraction system (Dionex, Sunnyvale, CA). After removing the solvent, the samples were reconstituted, centrifuged (23,000 × g and 4 °C), and filtered.

Compounds of interest were separated with the Acquity UPLC system (Waters, Milford, MA) comprising a Waters BEH C18 column (2.1 × 100 mm). The gradient elution was completed with the conditions described earlier [1]. Distilled water containing 0.1% formic acid and acetonitrile were used as solvents. Analytes were detected with the negative ion mode of the Water TQD mass spectrometer employing single reaction monitoring. Two fragmentation reactions, with optimized collision energy and cone voltage, were recorded for each analyte and the internal standard. Calibration curves for each analyte were constructed from 0.3 to 35 µg/ml by successive dilutions of 1 mg/ml standard suspensions and analysed under the same conditions as for the samples. Data were acquired and processed with Waters MassLynx 4.1 SCN 919 software.

1. Rakoczy-Trojanowska M, Orczyk W, Krajewski P, Bocianowski J, Stochmal A, Kowalczyk M. ScBx gene based association analysis of hydroxamate content in rye (Secale cereale L.). J. Appl. Genet. 2017b; 58:1-9. https://doi.org/10.1007/s13353-016-0356-3
